# Global genomic surveillance strategy for pathogens with pandemic and epidemic potential 2022–2032

**DOI:** 10.2471/BLT.22.288220

**Published:** 2022-04-01

**Authors:** Lisa L Carter, M Anne Yu, Jilian A Sacks, Céline Barnadas, Dmitriy Pereyaslov, Sébastien Cognat, Sylvie Briand, Michael J Ryan, Gina Samaan

**Affiliations:** a Health Emergencies Programme, World Health Organization Lyon Office, 24 Rue Jean Baldassini, 69007 Lyon, France.; b Health Emergencies Programme, World Health Organization, Geneva, Switzerland.

The coronavirus disease 2019 (COVID-19) pandemic marked a breakpoint for genomic surveillance. The first severe acute respiratory syndrome coronavirus 2 (SARS-CoV-2) genetic sequences were shared on 10 January 2020, ten days after the World Health Organization (WHO) was notified of a cluster of pneumonia in China.^1^ Scientists across the globe immediately started developing countermeasures and the first diagnostic assay was made available on 13 January 2020.^1^^,^^2^ The speed of data sharing and pathogen characterization was unprecedented. Genomic surveillance was consistently used during the pandemic to monitor virus evolution and transmission, and to identify variants of concern that may impact countermeasures. 

During the pandemic, the International Health Regulations (2005) Emergency Committee for COVID-19 repeatedly recommended for State Parties to strengthen genomic surveillance strategies.^3^ The Independent Panel for Pandemic Preparedness and Response recently recommended regular funding for the delivery of specific global goods including genomic sequencing.^4^ In May 2021, through resolution 74.7, the World Health Assembly urged Member States to increase their capacity to detect new threats, including through laboratory techniques such as genomic sequencing.^5^ WHO began a process in July 2021 to develop a strategy to drive a unified vision on global genomic surveillance for pathogens with pandemic and epidemic potential.

WHO undertook a multistep consultative process to develop the strategy. Stakeholders consulted include global, regional and country networks, key partners such as philanthropies, donors, public health institutions, One Health partners, and WHO global and regional teams. Experts from global programmes such as polio, influenza, Ebola virus disease, antimicrobial resistance, human immunodeficiency virus and COVID-19 helped refine the strategy’s technical orientation considering the broader needs and the 10-year horizon.

WHO presented the draft strategy to Member States at a briefing on 25 November 2021 to ensure alignment with the mandate established through the resolution, followed by an online public consultation process to reach all stakeholders inclusively and transparently. A global online seminar engaging 800 stakeholders and hosted by WHO was conducted to raise awareness, present different stakeholder perspectives and encourage feedback. Nearly 100 sets of inputs from these consultations informed the final version of the strategy, launched in March 2022.^6^

The goal of the strategy is that genomic surveillance is strengthened and scaled for quality, timely and appropriate public health actions within local to global surveillance systems. Five key objectives contribute to this goal: (i) improve access to tools for better geographical representation; (ii) strengthen the workforce to deliver at speed, scale and quality; (iii) enhance data sharing and utility for streamlined local to global public health decision-making; (iv) maximize connectivity for timely value-add in the broader surveillance architecture; and (v) maintain a readiness posture.^5^

The strategy provides the foundation for implementation plans in line with these objectives. A set of core principles will guide implementation, with countries at the centre of the strategy, ensuring investments are targeted where they are most needed.

Multisectoral partnerships are critical to successful strategy implementation. Catalysed by the pandemic, networks at country, regional and global level have accelerated development of sequencing and bioinformatic capacity. Investment is needed to ensure sustained integration of these technologies into national public health systems, across human, animal and environmental health. The Group of Seven (G7) and G20 have advocated and raised political awareness about the game-changing opportunities for genomics.^7^^,^^8^ The Access to COVID-19 Tools (ACT) Accelerator demonstrated how bringing partners together can increase access to and equitable distribution of tools through capacity-building and promoting affordability and availability of platforms.^9^

WHO is the convenor and advocate for the strategy and will work with Member States and partners to implement the strategy. Global enablers and partnerships born out of the pandemic include the ACT Accelerator and the G7 International Pathogen Surveillance Network. Through its global presence, WHO will assist countries to contextualize genomics within the broader surveillance architecture, develop normative guidance and regulatory frameworks, provide tools and technical assistance to countries, establish research agendas and support capacity through training and simulation exercises.

The strategy’s development and the global spotlight on genomics has highlighted the importance of well-resourced and resilient end-to-end laboratory and surveillance systems. All countries should have access to sequencing, but access alone is not enough. The genomic surveillance value chain needs clear surveillance objectives, governance, foundational laboratory and data systems, linkages to regional and global networks, and coherence with complementary phenotypic, clinical and epidemiological characterization that together maximize data utility for effective public health action. Through implementation of this strategy, occurrence of pathogens with pandemic and epidemic potential can be detected earlier, thus reducing the severity of outbreaks and saving lives.
